# SARS during Pregnancy, United States

**DOI:** 10.3201/eid1009.040244

**Published:** 2004-09

**Authors:** Lauren J. Stockman, Sara A. Lowther, Karen Coy, Jenny Saw, Umesh D. Parashar

**Affiliations:** * Centers for Disease Control and Prevention, Atlanta, Georgia, USA;; † Santa Clara County Department of Public Health, San Jose, California, USA;; ‡ Private pediatric practice, San Jose, California, USA

**Keywords:** letter, SARS, pregnancy, transmission, breast milk

**To the Editor:** Two of eight persons with laboratory-confirmed severe acute respiratory syndrome–associated coronavirus (SARS-CoV) infection in the United States during 2003 were pregnant women. Robertson et al. (*1*) reported data describing one pregnant patient who recovered and delivered a healthy infant. We report data concerning the second patient, with follow-up 1 month after the child's birth.

The patient, a healthy, 38-year-old woman in the 7th week of pregnancy, traveled with her husband to Hong Kong. From March 1 to March 6, 2003, they stayed at the Hong Kong hotel where it is believed a physician from China spread SARS-CoV to several guests. These guests were the index case-patients for subsequent outbreaks in Hong Kong, Vietnam, Singapore, and Toronto, Canada (*2*). The woman and her husband returned to the United States on March 6; the husband had onset of SARS illness on March 13. On March 19, the patient had onset of an illness with fever (temperature 37.8–40°C), muscle aches, chills, headache, runny nose, productive cough, wheezing, and shortness of breath. A chest radiograph showed a diffuse infiltrate in the left lung. The patient was hospitalized for 9 days and given broad-spectrum antimicrobial drugs. She recovered from her illness, and enzyme immunoassay and immunofluorescent assays conducted on serum samples on days 28 and 64 after illness onset were positive for antibodies to SARS-CoV.

The patient had an uneventful pregnancy until the last trimester, when her blood glucose levels were elevated. Early spontaneous rupture of membranes initiated preterm labor, and a cesarean section was performed at 36 weeks' gestation because of fetal distress. A 5-pound, 7-ounce, healthy boy was delivered without complications. Apgar scores were 7 at 1 minute and 8 at 5 minutes. The newborn had no illness, abnormalities, or congenital malformations. Serum samples from the patient at delivery were positive for antibodies to SARS-CoV, but cord blood and placenta samples were negative. Breast milk samples on postpartum days 12 and 30 were also negative for SARS-CoV antibodies. Blood, stool, and nasopharyngeal swab samples from the patient and cord-blood samples showed no viral RNA by reverse transcription–polymerase chain reaction. Stool samples from the newborn, collected on days 12 and 30 after delivery, were also negative for viral RNA.

Although other countries have reported cases of severe illness and poor outcome associated with SARS-CoV infection during pregnancy (*3**–**5*), neither of the two pregnant SARS case-patients in the United States had serious adverse outcomes. The presence of antibodies to SARS-CoV in breast milk might be influenced by the time of infection in relation to gestation. Robertson et al. (*1*) reported that antibodies to SARS-CoV were detected in the breast milk of a patient who was infected at 19 weeks' gestation; however, the patient in this case was infected at 7 weeks' gestation, and antibodies to the virus were not detected in her breast milk. No reports have indicated vertical transmission of SARS-CoV, a finding that is supported by our data. However, too few cases have been studied to clearly define the risks and provide guidance for treating pregnant women infected with SARS CoV.
